# Obstetrics and gynecology resident perception of virtual fellowship interviews

**DOI:** 10.1186/s12909-022-03113-3

**Published:** 2022-01-25

**Authors:** Jia Jennifer Ding, Phinnara Has, B. Star Hampton, Dayna Burrell

**Affiliations:** 1 Department of Obstetrics and Gynecology, Yale School of Medicine, 333 Cedar St., P.O. Box 208063, New Haven, CT 06520-8063 USA; 2 Lifespan Biostatistics, Epidemiology and Research Design, Rhode Island Hospital, Warren Alpert Medical School of Brown University, Providence, RI USA; 3 Department of Obstetrics and Gynecology, Women and Infants Hospital, Alpert Medical School of Brown University, Providence, RI USA

**Keywords:** OB/GYN fellowship application, Virtual interviews, COVID-19 pandemic

## Abstract

**Background:**

Travel restrictions amidst the COVID-19 pandemic reshaped interviewing for fellowships into a predominantly virtual process. How this impacts Obstetrics and Gynecology (OB/GYN) resident approaches to fellowship application and Match navigation is largely unknown.

**Methods:**

We performed a cross-sectional survey study of fourth year OB/GYN residents in the United States who participated in at least one virtual fellowship interview in 2020. We collected information regarding demographics, application strategy, perceived strengths and weaknesses of virtual interviews, and confidence with rank list creation. Descriptive statistics were used for categorical variables and responses pre- and post-Match were compared using Fisher’s exact test.

**Results:**

Seventy-five out of an estimated 490 applicants (~ 15% response rate) completed the survey. Of the respondents, 65.3% felt they interviewed at more programs virtually than they would anticipate completing in person, but perceived less confidence in having the necessary information (*n* = 45, 60%) or understanding the culture of programs (*n* = 59, 78.7%) to create a rank list. Cost savings were the main benefit of virtual interviews (*n* = 50, 66.7%), and inability to get a true “feel” for a program was the biggest limitation (*n* = 43, 57.3%). A majority (46.7%) advocate for a future hybrid interview process.

**Conclusions:**

OB/GYN residents pursuing fellowship reported interviewing at more programs during the virtual season, but had less confidence with rank list creation. Cost savings benefits are weighed against difficulty getting a “feel” for programs virtually. Most would advocate for a future hybrid interview process.

**Supplementary Information:**

The online version contains supplementary material available at 10.1186/s12909-022-03113-3.

## Background

Although restrictions imposed by the COVID-19 pandemic changed the process of Obstetrics and Gynecology (OB/GYN) subspecialty fellowship recruitment to an exclusively virtual experience for the 2020 recruitment season, some have long questioned the necessity and cost of traditional in-person interviews [1].

In a 2014 survey of first-year Maternal-Fetal Medicine fellows, the cost of in-person interviews amounted to almost $7000 U.S. dollars per applicant. This monetary cost did not include the toll associated with the physical burden of travel and time away from clinical duties, which often includes a complex system of arranging clinical coverage. Previously, programs have made small steps toward “regionalization” of interviews (offering sequential interview dates for geographically grouped programs) and have considered the role of virtual interviews and the idea of a centralized “job fair” (where fellowship administrative personnel gather in one place to meet with a crowd of applicants), but innovation has been slow without impetus [2].

However, while financial and logistical advantages of virtual interviewing may be obvious, OB/GYN resident perception of virtual fellowship interviews, strengths and limitations of virtual interviewing, and the impact on creating rank lists is largely unknown [3]. Furthermore, best practices regarding virtual interview implementation and execution to optimize information and culture conveyance remain unexplored. Lessons learned from OB/GYN resident perception of virtual fellowship interviews may help improve future residency and fellowship virtual interview experience.

The aim of this study is to assess OB/GYN resident strategies in fellowship application, perception of the strengths and limitations of virtual fellowship interviews, and overall confidence with decision-making in creating their rank list.

## Methods

We performed an anonymous cross-sectional survey study of fourth-year OB/GYN residents in the United States applying into subspecialty fields including Family Planning, Female Pelvic Medicine and Reconstructive Surgery, Gynecologic Oncology, Maternal-Fetal Medicine, Minimally Invasive Gynecologic Surgery, Pediatric and Adolescent Gynecology, and Reproductive Endocrinology and Infertility. This novel de-identified electronic survey consisted of 39 multiple choice, Likert scale, and free response questions covering demographics, strategies regarding number of programs targeted, perception of programs based on pre-review materials provided, self-perception of performance during the virtual interview day, preferences regarding the practical implementation of virtual interviews, confidence with decision-making in rank-list creation, and overall perception of virtual interviews (see Additional file 1 for survey questionnaire). In the absence of a validated survey tool, our novel survey was created by an experienced education team comprising of an OB/GYN Residency Program Director and Academic Chief Resident, and this survey incorporated feedback from Chief Residents applying into fellowships of multiple subspecialties as well as Fellowship Directors all from one academic OB/GYN Residency Program in the Northeast. Respondents were asked to provide perceptions of the virtual experience as compared to an otherwise anticipated in-person interview season, acknowledging prior experience with in-person interviews for residency. The survey concluded with an open response question to allow respondents to share additional information on the virtual interview experience. This voluntary 10-min survey was disseminated via electronic mail to all residency program directors in the American College of Obstetrics and Gynecology (ACOG) directory (which includes programs in all fifty states and Canada) and requested to be forwarded to fourth-year residents within the program who completed at least one virtual fellowship interview. We estimated 490 applicants to fellowships in OB/GYN based on data reported by the National Resident Matching Program (NRMP) 2019 Specialties Match [4]. The NRMP is a program in the United States that has been in place since 1952 whereby medical students and resident physicians as well as training institutions can “rank” each other, and a computer algorithm maximizes the most desired matches between candidates and programs. We offered ten randomly selected participants a twenty-dollar e-gift card as incentive for completing the survey. Only completed surveys were analyzed. The survey was open from October 6, 2020 to October 25, 2020. Two reminders for survey completion were sent via electronic mail during this period to the same initial group of OB/GYN Residency Program Directors. Responses were recorded in a secure Research Electronic Data Capture database (REDCap, Vanderbilt University) designed to securely capture electronic data for research. Descriptive statistics were utilized for categorical variables. An unplanned secondary analysis comparing responses before Match (October 14, 2020 at noon EST) with those after Match was completed, and Fisher’s exact test was used to compare pre-Match and post-Match cohort categorical variables. Two-tailed *p*-values were calculated and the significance level was set at < 0.05. Open-answer questions responses were reviewed for themes. The study was approved as exempt by the Institutional Review Board at Women and Infants Hospital in Providence, Rhode Island.

## Results

A total of 75 surveys were completed yielding an estimated 15% response rate based on 2019 NRMP fellowship applicant data. Of 75 completed surveys, the majority of respondents were White female-identifying candidates from academic programs in the Northeast (Table 1). Most respondents applied into Maternal-Fetal Medicine (*n* = 29, 38.7%) or Gynecologic Oncology (*n* = 19, 25.3%). Among respondents, 64% completed the survey prior to the Match, while 36% completed it post-Match. No respondents reported past experience with virtual interviews. Though most respondents reported applying to the same number of programs as they presumed they would have for in-person interviews (*n* = 57, 76.0%), most respondents reported interviewing at more programs than they anticipated they would have for in-person interviews (*n* = 49, 65.3%), citing both minimal additional cost and anticipation of a more difficult year matching as reasons for doing so. Among respondents, 48.0% estimated saving at least $5000 U.S. dollars on travel expenses, and 95.9% reported anticipating the need for more coverage with in-person as compared to virtual interviews (Table 2).

**Table 1:** Demographic data for Ob/Gyn Fellowship applicant survey respondents

*N* = 75	N (%)
How would you characterize your residency program?	
Academic-university based	64 (85.3)
Community	3 (4.0)
Community-Academic affiliated	8 (10.7)
Geographic region in US is residency program located	(*n* = 74)
Northeast	31 (41.9)
Northwest	8 (10.8)
Midwest	12 (16.2)
Southeast	12 (16.2)
Southwest	7 (9.5)
Outside continental US	4 (5.4)
Gender	
Female	63 (84.0)
Male	12 (16.0)
Race/Ethnicity*	
White	49 (65.3)
Black	4 (5.3)
Hispanic	4 (5.3)
Asian	17 (22.7)
Other	4 (5.3)
Age	
26-30	41 (54.7)
31-35	29 (38.7)
> 35	5 (6.7)

*Does not sum to 100%

**Table 2:** Fellowship interview season data

*N* = 75	N(%)
**Applicant Residency Program background**	
Percentage of residents from your program who pursue fellowship:	
< 25%	13 (17.3)
25-50%	25 (33.3)
51-75%	21 (28.0)
> 75%	16 (21.3)
Which fellowships does your institution offer?*	
Family Planning	24 (32.0)
Female Pelvic Medicine and Reconstructive Surgery	37 (49.3)
Gynecologic Oncology	47 (62.7)
Maternal-Fetal Medicine	57 (76.0)
Minimally Invasive Gynecologic Surgery	23 (30.7)
Pediatric and Adolescent Gynecology	5 (6.7)
Reproductive Endocrinology and Infertility	37 (49.3)
None	6 (8.0)
**Application**	
Which fellowship did you apply for?	
Family Planning	9 (12.0)
Female Pelvic Medicine and Reconstructive Surgery	3 (4.0)
Gynecologic Oncology	19 (25.3)
Maternal-Fetal Medicine	29 (38.7)
Minimally Invasive Gynecologic Surgery	8 (10.7)
Pediatric and Adolescent Gynecology	1 (1.3)
Reproductive Endocrinology and Infertility	6 (8.0)
Did you Match in the fellowship you applied for?	
Yes	27 (36.0)
N/A; awaiting Match results	48 (64.0)
How many programs did you apply to?	
< 10	9 (12.0)
10-20	14 (18.7)
21-30	20 (26.7)
31-40	10 (13.3)
41-50	11 (14.7)
> 50	11 (14.7)
Virtual interviews affect number of programs applied to	
Applied to fewer	1 (1.3)
Applied to just as many	57 (76.0)
Applied to few more	14 (18.7)
Applied to significantly more	3 (4.0)
**Interviews**	
How many programs did you interview at	
1-5	7 (9.3)
5-10	12 (16.0)
11-20	37 (49.3)
21-30	18 (24.0)
31-40	1 (1.3)
Virtual interviews affect number of programs interviewed at	
Interviewed at fewer	3 (4.0)
Interviewed at just as many	23 (30.7)
Interviewed at few more	31 (41.3)
Interviewed at significantly more	18 (24.0)
Did you interview at more programs because:*	
Minimal cost accepting additional interviews	44 (58.7)
More difficulty matching due to virtual interviews	34 (45.3)
Other	7 (9.3)
How many programs did you rank	
1-5	11 (14.7)
5-10	11 (14.7)
11-20	41 (54.7)
21-30	11 (14.67)
31-40	1 (1.3)
How much did you save on travel this interview season	
< $500	6 (8.0)
$500-$1000	2 (2.7)
$1000-$3000	16 (21.3)
$3000-$5000	15 (20.0)
$5000-$7000	12 (16.0)
> $7000	24 (32.0)
How many working days did you have to find coverage for	
1-5	32 (42.7)
6-10	28 (37.3)
11-15	12 (16.0)
16-20	3 (4.0)
How would this be different if attended in-person interviews	(*n* = 74)
Would’ve needed fewer days covered	1 (1.4)
Would’ve needed same number of days covered	2 (2.7)
Would’ve needed more days covered	71 (95.9)
Did you have to use vacation days for interviews	
Yes	27 (36.0)
No	48 (64.0)
Did you have prior experience interviewing on virtual platform	
Yes	0 (−-)
No	75 (100.0)
**Perception of the program**	
Pre-interview materials	
Very helpful	31 (41.3)
Somewhat helpful	37 (49.3)
Neutral	3 (4.0)
Not very helpful	3 (4.0)
N/A	1 (1.3)
Program brochure	
Very helpful	16 (21.3)
Somewhat helpful	34 (45.3)
Neutral	14 (18.7)
Not very helpful	4 (5.3)
Not helpful at all	1 (1.3)
N/A	6 (8.0)
Program video	
Very helpful	34 (45.3)
Somewhat helpful	31 (41.3)
Neutral	7 (9.3)
Not helpful at all	1 (1.3)
N/A	2 (2.7)
Program website	
Very helpful	25 (33.3)
Somewhat helpful	37 (49.3)
Neutral	9 (12.0)
Not very helpful	2 (2.7)
Not helpful at all	2 (2.7)
Program reputation	
Very helpful	36 (48.0)
Somewhat helpful	31 (41.3)
Neutral	7 (9.3)
Not very helpful	1 (1.3)
Program social media	
Very helpful	8 (10.7)
Somewhat helpful	21 (28.0)
Neutral	29 (38.7)
Not very helpful	8 (10.7)
Not helpful at all	4 (5.3)
N/A	5 (6.7)
Personalized interview schedule	
Very helpful	36 (48.0)
Somewhat helpful	30 (40.0)
Neutral	3 (4.0)
Not very helpful	3 (4.0)
N/A	3 (4.0)
Pre-interview “social” or “happy hour”	
Very helpful	15 (20.0)
Somewhat helpful	31 (41.3)
Neutral	10 (13.3)
Not very helpful	6 (8.0)
Not helpful at all	6 (8.0)
N/A	7 (9.3)
Interview day program overview information	
Very helpful	45 (60.0)
Somewhat helpful	25 (33.3)
Neutral	3 (4.0)
Not very helpful	1 (1.3)
N/A	1 (1.3)
Interview day interviews	
Very helpful	43 (57.3)
Somewhat helpful	30 (40.0)
Neutral	1 (1.3)
Not very helpful	1 (1.3)
Fellows Q&A	(*n* = 74)
Very helpful	44 (59.5)
Somewhat helpful	24 (32.4)
Neutral	5 (6.8)
N/A	1 (1.4)
Informational video/virtual tour	
Very helpful	19 (25.3)
Somewhat helpful	37 (49.3)
Neutral	12 (16.0)
Not very helpful	5 (6.7)
N/A	2 (2.7)
**Preference with specifics aspects of virtual interview**	
What virtual interview platform did you prefer*	
ZOOM	75 (100)
WEBEX	7 (9.3)
Microsoft Teams	9 (12.0)
Eposterboard	3 (4.0)
Other	1 (1.3)
For virtual interviews, would you prefer interviewing with	
Individual interviewer	66 (88.0)
Panel of interviewers	9 (12.0)
What is your preferred length of virtual interview	(*n* = 75)
15 min	23 (30.7)
18 min	8 (10.7)
20 min	42 (56.0)
25 min	2 (2.7)
Preferred number of interviews within an interview day	
Mean (SD)	4.9 (0.9)
Median (Min-Max)	5 (3-8)
IQR (Q1-Q3)	(4-5)
Preferred amount of break time between virtual interviews	
2 min	7 (9.3)
5 min	57 (76.0)
10 min	11 (14.7)
In between interviews, prefer meeting group or time away?	
Group break room	3 (4.0)
Time to myself	28 (37.3)
Combination of group break room and time to myself	44 (58.7)

*Does not sum to 100%

Regarding individual components of the fellowship interview experience, when rated on a Likert scale (ranging from very helpful, somewhat helpful, neutral, not very helpful, not helpful at all), respondents mostly rated the program video (*n* = 34, 45.3%), program reputation (*n* = 36, 48.0%), personalized interview schedule (*n* = 36, 48.0%), interview day program overview (*n* = 45, 60.0%), interview day interviews (*n* = 43, 57.3%), and “question and answer (Q&A)” session with fellows (*n* = 44, 59.5%) as *very helpful*, whereas pre-review materials (*n* = 37, 49.3%), program brochure (*n* = 34, 45.3%), program website (*n* = 37, 49.3%), and informational video/virtual tour (*n* = 37, 49.3%) were only *somewhat helpful*. The program “social” or “happy hour” was rated as *somewhat helpful* by the majority of applicants (*n* = 31, 41.3%). Program social media was the only field to be rated largely *neutral* (*n* = 29, 38.7%). All participants reported preference with the Zoom platform for interviewing, and most (*n* = 66, 88.0%) preferred interviewing with individuals rather than a panel of interviewers (defined as a group comprised of two or more interviewers). When asked about the logistics of an interview day, most respondents preferred an average of five 20-min interviews (*n* = 42, 56.0%) with a 5-min break in between each interview (*n* = 57, 76.0%) (Table 2).

Regarding perception of self-performance and confidence with rank list decision-making, the majority of respondents felt they were able to effectively convey their strengths and sense of personality (*n* = 69, 92.0% and *n* = 65, 85.3% respectively), but were less confident in having the necessary information (*n* = 45, 60%) or understanding the culture of various programs (*n* = 59, 78.7%) to create an informed rank list, citing inability to get a true “feel” for the program in the majority of instances (*n* = 43; 57.3%) (Table 3). When asked their preference for interview delivery, 13.3% preferred an exclusively in-person interview process, with 40.0% preferring an all-virtual experience and 46.7% advocating for a hybrid interview process of in-person and virtual interviews. Travel cost savings were cited as the main benefit of virtual interviews (*n* = 50, 66.7%), and inability to get a true “feel” for a program was identified as the biggest limitation of the virtual interview season (*n* = 43, 57.3%) (Table 4).

**Table 3:** Perception of self-performance and confidence with decision-making

*N* = 75	N(%)
I was able to effectively communicate my strengths	
Strongly disagree	3 (4.0)
Disagree	2 (2.7)
Neutral	1 (1.3)
Agree	46 (61.3)
Strongly agree	23 (30.7)
I was able convey a sense of my personality	
Strongly disagree	4 (5.3)
Disagree	3 (4.0)
Neutral	4 (5.3)
Agree	45 (60.0)
Strongly agree	19 (25.3)
Compared to in-person, how confident did you feel about having the information you need to make informed decision for rank list?	
Much less confident	6 (8.0)
Somewhat less confident	39 (52.0)
Equally confident	29 (38.7)
Much more confident	1 (1.3)
Compared to in-person, how confident did you feel about understanding culture of various programs to make informed decision for rank list?	
Much less confident	14 (18.7)
Somewhat less confident	45 (60.0)
Equally confident	15 (20.0)
Much more confident	1 (1.3)

**Table 4:** Overall perception of virtual interviews

*N* = 75	N(%)
If you had to repeat this interview season, would you:	
Prefer to repeat virtual interview	30 (40.0)
Prefer all in-person interview	10 (13.3)
Advocate for hybrid interview process	35 (46.7)
What do you consider the greatest benefit of virtual interviews	
Travel expenses saved	50 (66.7)
Time saved	10 (13.3)
Convenience	13 (17.3)
Other	2 (2.7)
What do you consider the biggest limitation to virtual interviews	
Inability to get a true “feel” for a program	43 (57.3)
Technological difficulties	2 (2.7)
Not being able to meet fellow interviewees and connect in person	14 (18.7)
Not visiting the physical location of the program	14 (18.7)
None	2 (2.7)

In our unplanned secondary analysis, when comparing participants who responded to our survey before and after Match, there were no significant differences observed in interview format preference (*p* = 0.89), or overall confidence in decision making to inform a rank list (*p* = 1.00) (Table 5).

**Table 5:** Comparisons by pre-Match vs. post-Match respondents

*N* = 75	pre-Match (*n* = 52)	post-Match (*n* = 23)	*p*-value^1^
Which interview format would you prefer in the future?			
In-person interview	11 (21.2)	4 (17.4)	0.89
Virtual interview	19 (36.5)	8 (34.8)	
Initial virtual followed by selective in-person	22 (42.3)	11 (47.8)	
Compared to in-person, how confident did you feel about having the information you need to make informed decision for rank list?			
Much less confident	4 (7.7)	2 (8.7)	1.00
Somewhat less confident	27 (51.9)	12 (52.2)	
Equally confident	20 (38.5)	9 (39.1)	
Much more confident	1 (1.9)	0 (−-)	
Compared to in-person, how confident did you feel about understanding culture of various programs to make informed decision for rank list?			
Much less confident	12 (23.1)	2 (8.7)	0.43
Somewhat less confident	30 (57.7)	15 (65.2)	
Equally confident	9 (17.3)	6 (26.1)	
Much more confident	1 (1.9)	0 (−-)	
Having participated in an exclusively virtual interview season, would you recommend future applicants			
Apply for fewer number of programs as in-person interviews	1 (1.9)	0 (−-)	0.69
Apply for the same number of programs as in-person interviews	38 (73.1)	19 (82.6)	
Apply for more number of programs as in-person interviews	13 (25.0)	4 (17.4)	

^1^Fisher’s exact test

## Discussion

In our study of fourth-year OB/GYN residents in the United States applying into subspecialty fellowships via a virtual interview process during the COVID-19 pandemic in 2020, we found that although candidates applied to about as many programs as they anticipated they would have for in-person interviews, participants were more likely to interview at more programs due to minimal additional cost in the virtual format as well as perceived difficulty in matching during an unprecedented year. Respondents identified program interview components including a pre-recorded video of the program and Q&A with current fellows as very helpful to the process, whereas events such as the virtual “happy hour” and social media presence of the program were surprisingly less helpful. Additionally, despite interviewing at more programs, respondents reported feeling less confident in their decision-making when creating a rank list compared with perceived confidence had the interview season been conducted in person. Inability to get a true “feel” for each program was cited as the biggest limitation of the virtual interview process, with programs blending into each other and losing individuality on a virtual platform, while cost savings, convenience and minimizing burden of clinical coverage were identified as potential advantages. Importantly, a common theme among free response answers was the increased access to interviews, especially for applicants from smaller programs who would have had more difficulty arranging for clinical coverage given that less time overall was needed for interviews. Virtual interviews may therefore offer increased accessibility and equity as compared to in-person interviews. Nevertheless, more respondents would rather repeat an entirely virtual than in-person interview experience, although most would advocate for a hybrid process of virtual and in-person interactions, which may combine the advantages of virtual interviews without missing out on irreplaceable aspects of in-person interviews.

Our results supplement scant existing though expanding knowledge regarding conceptualization of virtual interview processes within OB/GYN by both applicants and interviewers. A recent study by Lewkowitz et al. of Maternal-Fetal Medicine fellowship applicants suggested that OB/GYN residents are likely to accept more virtual interview offers than in-person interview offers given increased ease and convenience, which may have unintended downstream consequences on the ability of certain candidates to be offered interview opportunities [5]. Results from our study are in line with these findings, and suggest that it may be prudent to trial a system where applicants are limited in terms of number of accepted interviews to ensure that all qualified applicants are given an opportunity to interview at programs of genuine interest. Further, although applicants may view virtual interviews positively, especially when travel and time costs are considered [5], our study demonstrates that applicants may weary of fully virtual interviews given limitations in ability to accrue meaningful information regarding each program and inability to get a sense of less concrete markers such as overall program culture and feel. While some component of virtual residency and fellowship interviews may be here to stay, it is of utmost importance to investigate the pitfalls of these changes to ensure applicants feel empowered and advocated for during this process.

Our study was limited by an inability to compare groups in real-time, as respondents were asked to compare their virtual interview experience with a hypothetical comparison of an in-person interview experience. Respondent views relied on perceptions having interviewed in person for residency in the recent past. Also, despite our attempt to reach all applicants by disseminating the survey to all program directors in the ACOG program director database several times, we cannot be certain all OB/GYN subspecialty applicants had access to respond to our survey. Applicants who are not current U.S. or Canadian OB/GYN residents (such as practicing generalists, as well as other international applicants) were also not privy to participate in our study. Our response rate of ~ 15% is a substantial limitation of our study. Furthermore, although most OB/GYN residents in the United States identify as White and female (62.7 and 83.8% respectively according to the 2020 report on residents from the Association of American Medical Colleges [6]) and while the majority of our respondents identified as White females from northeast academic residency programs, this portrays only a proportion of voices among agents invested in the fellowship Match progress and limits the generalizability of our results. This highlights the importance of repeating this study and reaching more respondents in order to collect more representative data and allow for sub-analysis such as by specialty field or geographic region.

Our study poses a substantial question for subsequent interview cycles: how can hybrid application processes be implemented with maximal acceptance and trust by all stakeholders? Options previously proposed include having candidates choose between virtual or in-person interviews with programs offering both options versus an initial round of virtual interviews followed by a subsequent in-person visit to the program for interested parties. In-person visits can even be scheduled after programs have submitted their Rank list and prior to applicants finalizing their Rank list to help applicants evaluate intangible elements such as culture of programs without programs taking these in-person visits into account, as not all applicants will be able to afford traveling to every program. Future interview formats should take into consideration cost of any in-person component as these savings are a major advantage of the virtual process. Further study dedicated to creating and implementing best practices for virtual interview components and consideration of a hybrid interview model combining virtual and subsequent in person events are essential next steps. This information is crucial to both administrators and applicants in coming years as we plan for a new evaluation landscape that is both more streamlined and personal, one that minimizes unnecessary time commitment and cost while enhancing both stakeholders’ ability to evaluate the other party without bias.

## Conclusions

Our study demonstrated certain advantages and drawbacks of an exclusively virtual interview process, mainly the tradeoff of ease in scheduling interviews and cost savings for the uncertainty of ranking a program highly after only interacting in a virtual space. Respondents indicated the desire to consider a hybrid of virtual and in-person formats for future interview seasons.

## Supplementary Information

**Additional file 1.**

## Data Availability

All data generated or analyzed during this study are included in this published article and its supplementary information files.

## Abbreviations

ACOG: American College of Obstetricians and Gynecologists
AAMC: Association of American Medical Colleges
COVID-19: Coronavirus disease of 2019
NRMP: National Resident Matching Program
OB/GYN: Obstetrics and Gynecology
Q&A: Question & Answer
REDCap: Research Electronic Data Capture Database
