# Supplementary material for: Obstetrics and gynecology resident perception of virtual fellowship interviews
Source: BMC Med Educ. 2022 Jan 25;22:58. doi: 10.1186/s12909-022-03113-3 (PMC8789205; doi:10.1186/s12909-022-03113-3)
Supplement: Supplementary file 1 — Additional file 1. [file 12909_2022_3113_MOESM1_ESM.docx]

**Supplemental Materials**

**Survey questionnaire**

**Section 1: Demographics**

1. How would you characterize your residency program?
   1. Academic-University based
   2. Community
   3. Community-Academic affiliated
2. In what geographic region in the United States is your residency program located?
   1. Northeast
   2. Northwest
   3. Midwest
   4. Southeast
   5. Southwest
   6. Outside continental US
3. I identify as
   1. Female
   2. Male
   3. Non-gender binary
   4. Prefer not to answer
4. How would you describe yourself (choose all that apply)
   1. White
   2. Black or African American
   3. Hispanic
   4. Asian
   5. American Indian or Alaskan Native
   6. Native Hawaiian or Pacific Islander
   7. Other
5. My age is
   1. <20
   2. 20-25
   3. 26-30
   4. 31-35
   5. 36-40
   6. >40
6. What percentage of the residents from your training program go on to pursue a fellowship (Minimally Invasive Gynecologic Surgery, Gynecologic Oncology, Maternal Fetal Medicine, Female Pelvic Medicine and Reconstructive Surgery, Family Planning, Pediatric and Adolescent Gynecology, or Reproductive Endocrinology and Infertility)?
   1. <25%
   2. 25-50%
   3. 51-75%
   4. >75%
   5. Unsure
7. Which of the following fellowships does your institution offer (choose all that apply)?
   1. Family Planning (FP)
   2. Female Pelvic Medicine and Reconstructive Surgery (FPMRS)
   3. Gynecologic Oncology (Gyn Onc)
   4. Maternal-Fetal Medicine (MFM)
   5. Minimally Invasive Gynecologic Surgery (MIGS)
   6. Pediatric and Adolescent Gynecology (PAGS)
   7. Reproductive Endocrinology and Infertility (REI)
   8. Unsure
   9. None
8. Which fellowship did you apply for?
   1. Family Planning (FP)
   2. Female Pelvic Medicine and Reconstructive Surgery (FPMRS)
   3. Gynecologic Oncology (Gyn Onc)
   4. Maternal-Fetal Medicine (MFM)
   5. Minimally Invasive Gynecologic Surgery (MIGS)
   6. Pediatric and Adolescent Gynecology (PAGS)
   7. Reproductive Endocrinology and Infertility (REI)
9. Did you Match in the fellowship you applied for?
   1. Yes
   2. No
   3. Not applicable, awaiting Match results

Section 2: Application Process

1. How many programs did you apply to?
   1. < 10
   2. 10-20
   3. 21-30
   4. 31-40
   5. 41-50
   6. >50
2. How did virtual interviews affect the number of programs you applied to?
   1. I applied to fewer programs than I would have for in-person interviews
   2. I applied to just as many programs as I would have for in-person interviews
   3. I applied to a few more programs as I would have for in-person interviews
   4. I applied to significantly more programs as I would have for in-person interviews
3. How many programs did you interview at?
   1. 1-5
   2. 5-10
   3. 11-20
   4. 21-30
   5. 31-40
   6. >40
4. How did virtual interviews affect the number of programs you interviewed at?
   1. I interviewed at fewer programs than I would have for in-person interviews
   2. I interviewed at just as many programs as I would have for in-person interviews
   3. I interviewed at a few more programs as I would have for in-person interviews*
   4. I interviewed at significantly more programs as I would have for in-person interviews*
5. (Branching question from question 13 if “c” or “d” were selected) Did you interview at more programs because (select all that apply):
   1. There was minimal cost associated with accepting each additional interview
   2. You perceived more difficulty matching this year due to virtual interviews and wanted to expand your rank list
   3. Other _____
6. How many programs did you rank?
   1. 1-5
   2. 5-10
   3. 11-20
   4. 21-30
   5. 31-40
   6. >40
7. How much would you estimate you saved on travel expenses this interview season?
   1. <$500
   2. $500-$1000
   3. $1000-$3000
   4. $3000-$5000
   5. $5000-$7000
   6. >$7000
8. How many working days did you have to find coverage for this interview season?
   1. 1-5
   2. 6-10
   3. 11-15
   4. 16-20
   5. 21-25
   6. >25
9. How would this be different if you attended in-person interviews?
   1. I would have needed fewer days covered
   2. I would have needed the same number of days covered
   3. I would have needed more days covered
10. Did you have to use vacation days for interviews?
    1. Yes
    2. No
11. Did you have prior experience interviewing on a virtual platform?
    1. Yes
    2. no

**Section 3: Perception of the Program**

1. Please rank the following pre-interview materials and information in terms of how helpful they were to prepare for the interview and get a sense of the program: 1=Very helpful, 2= Somewhat helpful, 3=Uncertain, 4=Not very helpful, 5=Not helpful at all 6= Not applicable (NA)

| Pre-interview materials | 1= Very helpful | 2= Somewhat helpful | 3= Uncertain | 4=Not very helpful | 5= Not helpful at all | 6= NA |
| --- | --- | --- | --- | --- | --- | --- |
| Program brochure |  |  |  |  |  |  |
| Program video |  |  |  |  |  |  |
| Program website |  |  |  |  |  |  |
| Program reputation |  |  |  |  |  |  |
| Program social media |  |  |  |  |  |  |
| Personalized interview schedule |  |  |  |  |  |  |

1. Please comment on any additional materials you received and how you would rank them on the following scale (1= Very helpful, 2= Somewhat helpful, 3=Uncertain, 4=Not very helpful, 5=Not helpful at all 6= Not applicable): _____
2. Please rank the following elements of the interview process in terms of how helpful they were to allow you to get a sense of the program: 1=Very helpful, 2= Somewhat helpful, 3=Uncertain, 4=Not very helpful, 5=Not helpful at all 6= Not applicable (NA)

| Interview element | 1= Very helpful | 2= Somewhat helpful | 3= Uncertain | 4=Not very helpful | 5= Not helpful at all | 6= NA |
| --- | --- | --- | --- | --- | --- | --- |
| Pre-interview “social” or “happy hour” |  |  |  |  |  |  |
| Interview day program overview information |  |  |  |  |  |  |
| Interview day interviews |  |  |  |  |  |  |
| Fellows Q&A |  |  |  |  |  |  |
| Program website |  |  |  |  |  |  |
| Informational video/virtual tour |  |  |  |  |  |  |

1. Please include any additional interview elements you experienced and how you would rank them on the following scale (1= Very helpful, 2= Somewhat helpful, 3=Uncertain, 4=Not very helpful, 5=Not helpful at all 6= Not applicable): _____

Section 4: Perception of Self-Performance

1. The following questions are to understand your sense of your performance during the virtual interview process. Please rate your response with the following: 1= Strongly disagree, 2= Disagree, 3= Neutral, 4= Agree, 5= Strongly agree

| Question | 1=Strongly disagree | 2=Disagree | 3=Neutral | 4=Agree | 5=Strongly agree |
| --- | --- | --- | --- | --- | --- |
| I was able to effectively communicate my strengths |  |  |  |  |  |
| I was able convey a sense of my personality |  |  |  |  |  |

**Section 5: Preference with Specific Aspects of Virtual Interview**

1. What virtual interview platform did you prefer?
   1. ZOOM
   2. WEBEX
   3. Microsoft office
   4. Eposterboard
   5. Other: ____

1. After your recent virtual interview experience, which interview format would you prefer in the future?
   1. In-person interview
   2. Virtual interview
   3. Initial virtual interview followed by selective in-person interview
   4. Other: ____
2. For virtual interviews, would you prefer interviewing with ___
   1. Individual interviewer
   2. Panel of interviewers
3. What is your preferred length of virtual interview?
   1. 15 minutes
   2. 18 minutes
   3. 20 minutes
   4. 25 minutes
   5. 30 minutes
4. What is your preferred number of interviews?
   1. 3
   2. 4
   3. 5
   4. 6
   5. 7
   6. 8
   7. >8
5. What is your preferred amount of break time between virtual interviews?
   1. 2 minutes
   2. 5 minutes
   3. 10 minutes
   4. Other: ____
6. In between interviews, did you prefer meeting the group back in a virtual break room or having time away from your interview station?
   1. Group break room
   2. Time to myself
   3. A combination of group break room and time to myself

**Section 6: Confidence with Decision-Making**

For the following questions, please compare your experience in-person interviewing for residency with virtual interviewing for fellowship:

1. Compared to an in-person interview, how confident did you feel about having the information you need to make an informed decision for your rank list?
   1. Much less confident
   2. Somewhat less confident
   3. Equally confident
   4. Somewhat more confident
   5. Much more confident

1. Compared to an in-person interview, how confident did you feel about understanding the culture of various programs in order to make an informed decision for your rank list?
   1. Much less confident
   2. Somewhat less confident
   3. Equally confident
   4. Somewhat more confident
   5. Much more confident

**Section 7: Overall Perception of Virtual Interviews**

1. If you had to repeat this interview season, would you:
   1. Prefer to repeat virtual interview
   2. Prefer all in-person interview
   3. Advocate for hybrid interview process
2. What do you consider the greatest benefit of virtual interviews?
   1. Travel expenses saved
   2. Time saved
   3. Convenience
   4. None
   5. Other: ____
3. What do you consider the biggest limitation to virtual interviews?
   1. Inability to get a true “feel” for a program
   2. Technological difficulties
   3. Not being able to meet fellow interviewees and connect in person
   4. Not visiting the physical location of the program
   5. None
   6. Other: ____

**Section 8: Advice for Future Applicants**

1. Having participated in an exclusively virtual interview season, would you recommend future applicants
   1. Apply for fewer number of programs as in-person interviews
   2. Apply for the same number of programs as in-person interviews
   3. Apply for more number of programs as in-person interviews
2. Please feel free to share any additional information & thoughts on the virtual interview experience in the space below:
